# Cell-Based Therapies Used to Treat Lumbar Degenerative Disc Disease: A Systematic Review of Animal Studies and Human Clinical Trials

**DOI:** 10.1155/2015/946031

**Published:** 2015-05-14

**Authors:** David Oehme, Tony Goldschlager, Peter Ghosh, Jeffrey V. Rosenfeld, Graham Jenkin

**Affiliations:** ^1^The Ritchie Centre, Monash Institute of Medical Research (MIMR-PHI), Monash University, Clayton Branch, P.O. Box 6178, South Yarra, VIC 3141, Australia; ^2^Departments of Surgery and The Ritchie Centre, Monash Institute of Medical Research (MIMR-PHI), Monash University, Clayton Branch, 27-31 Wright Street, Clayton, VIC 3168, Australia; ^3^Proteobioactives Research Laboratories, P.O. Box 35, Brookvale, NSW 2100, Australia; ^4^Mesoblast Ltd, Melbourne, VIC 3000, Australia; ^5^Department of Surgery, Monash University, Clayton, VIC 3168, Australia; ^6^Department of Neurosurgery, Alfred Hospital, Level 5, The Alfred Centre, Commercial Road, Prahran, Melbourne, VIC 3168, Australia; ^7^The Ritchie Centre, Monash Institute of Medical Research (MIMR-PHI), Monash University, Clayton Branch, 27-31 Wright Street, Clayton, VIC 3168, Australia

## Abstract

Low back pain and degenerative disc disease are a significant cause of pain and disability worldwide. Advances in regenerative medicine and cell-based therapies, particularly the transplantation of mesenchymal stem cells and intervertebral disc chondrocytes, have led to the publication of numerous studies and clinical trials utilising these biological therapies to treat degenerative spinal conditions, often reporting favourable outcomes. Stem cell mediated disc regeneration may bridge the gap between the two current alternatives for patients with low back pain, often inadequate pain management at one end and invasive surgery at the other. Through cartilage formation and disc regeneration or via modification of pain pathways stem cells are well suited to enhance spinal surgery practice. This paper will systematically review the current status of basic science studies, preclinical and clinical trials utilising cell-based therapies to repair the degenerate intervertebral disc. The mechanism of action of transplanted cells, as well as the limitations of published studies, will be discussed.

## 1. Introduction

Low back pain is the leading cause of disability in the developed world and has an enormous social impact on patients and their families, as well as a devastating economic impact on healthcare budgets [1]. The annual cost of back pain in the United States is estimated to be as high as $500 billion [2]. Studies have shown that 75–80% of people will experience low back pain at some stage with a prevalence ranging from 15 to 45% [3]. Severe disc degeneration is associated with a twofold increase in chronic low back pain [4, 5]; however, despite this strong link between pain and disc degeneration [6–10], it is well recognised that not all patients with radiological evidence of disc degeneration will have symptoms. There are many potential pain generators in the lumbar spine in addition to the disc. Moreover, differentiating the ageing disc from the symptomatically degenerate disc remains a major challenge [11, 12].

The intervertebral disc (IVD) is a fibrocartilaginous articulation between adjacent vertebrae, which has a central hydrated gelatinous core, the nucleus pulposus (NP) surrounded by an outer fibrous-cartilaginous ring, the annulus fibrosis (AF), which consists of concentrically lamellated collagen fibres. Thin hyaline cartilage endplates attach the disc to the adjacent vertebral bodies and disc nutrition passes through these end plates to the predominantly avascular IVD [13, 14]. The IVD functions to facilitate movement and flexibility of the vertebral column, whilst also having the ability to recover from deformation following axial loading. The native cell population of the disc represents approximately 1% of the disc tissue but is pivotal in maintaining disc metabolism [13, 15]. Cells of the NP and inner AF demonstrate chondrocytic morphology whilst cells in the outer AF are more fibroblastic-like [16, 17].

The causes of disc degeneration are complex and multifactorial, including genetic, nutritional, and mechanical influences [17]. An imbalance between extracellular matrix degradation and synthesis results in progressive collapse and mechanical failure of the disc. An overall decrease in resident disc cell number and function and cellular responses to nutritional deficiencies leads to alterations in both the cartilaginous and proteoglycan matrix components of the disc [13, 18]. The loss of the pivotal water binding proteoglycan component leads to dehydration of the NP, impacting the discs ability to adequately distribute and recover from mechanical loading. As degeneration progresses neovascularisation, with concurrent neoinnervation, can occur within the degenerative AF and extend to within the NP [10, 19, 20], this pathological ingrowth of nerve fibres and vessels has been linked to the mechanical back pain experienced by patients with disc disease [10]. Endplate changes occur with thinning, calcification, and alterations in vascularity, as nutritionally deprived discs attempt to increase their nutrient supply. This creates a hostile environment that is a major challenge to maintain cell viability of both native cells or cells that are implanted in regenerative therapies. Moreover, changes within the adjacent vertebral bodies and endplates occur, including sclerosis and subchondral bone microfracture [13].

Whilst the degenerative cascade is well understood, low back pain can be a source of frustration for patients and clinicians due to difficulties in identifying the pain generator and a lack of treatment options available to successfully manage all patients. Initial treatments include conservative therapies such as analgesics, physical therapies, and psychological pain management strategies. When these nonoperative treatments fail, surgical interventions, such as lumbar fusion or total disc arthroplasty, are commonly performed. These treatments are often successful; however they do not address the underlying cause and, despite these interventions, some patients remain with chronic pain and disability.

Substantial progress has occurred in the fields of regenerative medicine, tissue engineering, and stem cell therapies, with the aim of treating and reversing disc degeneration, as well as augmenting and enhancing current treatments. Clinical trials have commenced utilising cell-based biological therapies to treat many common diseases, including those affecting the musculoskeletal system and, in particular, degenerative discopathies. Culture expanded disc chondrocytes and mesenchymal stem cells, isolated from bone marrow or other sources, are the two cell types most commonly used by researchers to biologically repair the degenerate disc. Other types of stem or progenitor cells, used in either an autologous or allogeneic fashion, have also been investigated in studies. Several small clinical trials have recently been published with another larger randomised phase-2 trial currently underway [21–24].

This paper will comprehensively review the current status of basic science studies as well as preclinical and clinical trials utilising cell-based therapies to repair the degenerate intervertebral disc. Significant positive and negative findings of trials published to date will be highlighted, and the relative benefits and limitations of various cell types and treatment strategies will be discussed. Animal models of disc degeneration and the applicability of these models to the human condition will also be addressed. Knowledge of what has been achieved to date, as well as the limitations of these achievements, is important to guide future trials as this exciting field of regenerative medicine translates toward the clinic.

## 2. Methods

**Figure 1:**
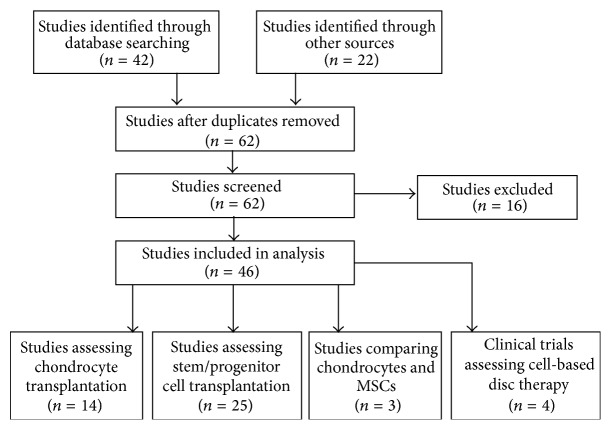
Flow diagram demonstrating the systematic analysis process.

We performed a literature search using the MEDLINE online electronic database between 1950 and 2013, Google Scholar, and the Cochrane Database. The following keywords were queried in combination with* intervertebral disc*:* stem cell*,* mesenchymal stem cell*,* progenitor cell*,* nucleus pulposus cell*,* disc chondrocyte*,* disc regeneration*, and* tissue engineering*. The search was limited to articles published in English. Studies utilising either stem cells, progenitor cells, or intervertebral disc chondrocytes to regenerate the intervertebral disc were included in the analysis. The indexes of suitable articles were reviewed for further relevant published studies. Publications comprised of* in vitro* work only were excluded. Studies were then grouped into one of the following four categories for analysis: (1) studies utilising chondrocyte transplantation, obtained from intervertebral disc tissue or other cartilage sources, (2) studies utilising stem and progenitor cell transplantation, including mesenchymal stem cells (MSCs) and other cell types obtained from noncartilaginous tissues, (3) studies comparing chondrocyte and stem cell transplantation, and (4) human clinical trials utilising any form of cell-based therapy to repair degenerative discs, including chondrocytes and stem cell therapies. The flow diagram for our search is outlined in Figure 1.

## 3. Results

### 3.1. Studies Utilising Intervertebral Disc or Chondrocyte (or Chondrocyte-Like) Cell Transplantation

There were 14 studies identified assessing the ability of disc derived and nondisc derived chondrocytes to regenerate IVDs, as shown in Table 1.

#### 3.1.1. Animal Models Utilised

2/14 studies utilised a rodent animal model (rat) [28, 33], whilst 12/14 studies utilised larger animal models (rabbit, canine, or monkey) [15, 25–27, 29–32, 34]. 12/14 studies utilised nucleotomy as the method of inducing degeneration, one study used a laser to damage the disc and, in the remaining study, a total discectomy was performed. It should be noted that the amount of nuclear material removed in the nucleotomy procedure differed between studies and is listed in Table 1.

#### 3.1.2. Cell Types Utilised

10/14 studies transplanted culture expanded NP cells into target discs. The remaining studies utilised AF cells, NP tissue, or whole disc. Allogeneic administration was performed in 10/14 studies and was autologous in 3/14, and xenogeneic administration was performed in one study, where human NP cells were injected into the rabbit disc [31]. 8/14 studies injected the cells into the target disc without a cell carrier, whilst 5 coadministered cells with either fibrin glue or another tissue engineered scaffold.

#### 3.1.3. Outcomes

In 12/14 studies (92%), in discs receiving cell treatment, degeneration was slowed or reversed on gross morphological or histological assessment and had increased matrix deposition, either proteoglycan or collagen, when compared to controls. 7/14 studies also demonstrated favourable radiological outcomes, being either preservation of disc height or increased T2 signal on MRI. 8 studies demonstrated viability of transplanted chondrocytes following injection, with Gruber et al. [28] demonstrating survival for up to 8 months following transplantation.

The study by Bertram et al. [25] showed that 90% of cells leaked out of the disc following injection in aqueous solution; however, this was reduced to 50% with fibrin glue coadministration. Luk et al. [32] found that whole disc transplant could be performed; however, despite surviving, the transplanted disc underwent severe degeneration. No other cell related morbidity or negative outcomes were reported in any other study utilising chondrocyte transplantation.

### 3.2. Studies Utilising Stem Cell and Progenitor Cell Transplantation

There were 25 studies assessing the ability of different types of stem cells or progenitor cells to regenerate the IVD identified, as shown in Table 2.

#### 3.2.1. Animal Models Utilised

6/25 studies used rodent animal models (rat or mouse), whilst 16/25 utilised larger animal models (rabbit canine, porcine, or ovine). 3/25 studies used normal nondegenerate discs. The remaining 22/25 studies used either needle puncture, nucleotomy, matrix degrading enzymes, annular injury, or axial loading to induce degeneration prior to the administration of cell therapy.

#### 3.2.2. Cell Type Utilised

Bone marrow derived MSCs were the most commonly used stem cell treatment, used in 20/25 (80%) studies [40, 41, 49, 52–54, 57–59, 61]. 9 of these studies used autologous administration of MSCs, 7/25 used allogeneic administration, and 4/25 studies utilised human MSC xeno-transplantation to treat degenerate animal discs. 3/25 studies used adipose derived MSCs, either autologous or xenogeneic human MSCs, whilst other cell types investigated included human embryonic stem cells (ESCs), autologous synovial derived MSCs, human olfactory neurosphere derived stem cells, and allogeneic mesenchymal precursor cells (MPCs). 3/25 studies utilised hyaluronic acid as the cell carrier, 5/25 studies used another hydrogel (hyaluronan,* Puramatrix*, or PFG-TGF-beta1) or fibrin based scaffold, whilst, in the remaining studies, the vehicle carrier was not defined and cells were presumably injected into discs in aqueous culture medium only.

#### 3.2.3. Outcomes

The outcomes of these studies are summarized in Table 2. 15/25 studies reported favourable radiological outcomes: either preservation of disc height or increased MRI T2 signal, following cell administration. 2/25 studies reported no improvement whilst the remaining studies did not specifically assess radiological outcomes. 14/25 studies demonstrated improved histological structure following cell transplantation, whilst 15/25 studies reported positive findings in terms of matrix restoration, utilising either total GAG or collagen II content or measuring expression of genes known to be important for matrix restoration, such as Col2a1, aggrecan, and Sox-9.

12/25 studies assessed the viability of cells following transplantation with varying survival times reported, ranging from 15 days to 48 weeks. Several other studies, however, reported leakage or nonviability of cells following injection. Omlor et al. [49] reported that only 9% of cells remained in the disc 3 days following implantation with fibrin glue, whilst Vadalà et al. [57] found no evidence of regeneration or the transplanted cells 9 weeks after intradiscal injection of allogeneic bone marrow MSCs.

### 3.3. Studies Comparing MSC and Chondrocyte Transplantation

Three studies directly compared the ability of MSCs and chondrocytes to regenerate IVDs [62, 63, 66], as shown in Table 3. Feng et al. [66] showed that autologous MSCs and NPCs were equivalent in maintaining disc height and MRI T2 signal, aggrecan and collagen II expression, and proteoglycan production. Allon et al. [63] found that transplantation of bilaminar coculture pellets of allogeneic MSCs and NPCs increased disc height and proteoglycan production. When used alone, however, both the MSCs and NPCs were equally ineffective in repairing the damaged rat disc.

Acosta et al. [62] found that nucleotomised porcine discs treated with allogeneic nondisc juvenile articular chondrocytes had increased glycosaminoglycan (GAG), DNA, and cartilage content compared to bone marrow derived MSCs. These allogeneic MSCs were found not to be viable at 3 months and there was no evidence of proteoglycan production in their model.

### 3.4. Clinical Trials Utilising Cell Based Disc Therapies

Four published clinical studies utilising cell-based therapies to treat human lumbar disc degeneration were identified [15, 22, 23, 65], as shown in Table 4. Three of these studies reported favourable results. The* EuroDISC* study, by Meisel and colleagues [15], investigated the percutaneous transplantation of autologous disc chondrocytes. Patients enrolled underwent a single level microdiscectomy procedure from which disc chondrocytes were harvested and expanded* in vitro* and subsequently injected into the NP three months postoperatively. Analysis at two years demonstrated that patients who received chondrocyte transplantation had significantly less back pain and increased NP T2 signal on MRI in both the treated and adjacent discs. Yoshikawa et al. [23] reported favourable outcomes following percutaneous intradiscal administration of autologous MSCs within collagen sponge in two elderly patients with degenerative disc disease [23]. At two years both patients demonstrated alleviation of both back and radicular symptoms. Orozco et al. [22] reported a pilot study of 10 patients with chronic low back pain and degenerative disc disease, again treated with percutaneously intradiscal administration of autologous MSCs. In this study, 90% of participants reported clinical benefit with significant decrease in pain and disability and improvement in quality of life.

Haufe and Mork [65] reported no improvement in clinical status following the transplantation of autologous, nonculture expanded, haematopoietic precursor stem cells (HSCs) into the discs of 10 patients. No patient demonstrated clinical improvement in back pain or disability. Notably, 85% of patients underwent surgery at the stem cell treated level at one year.

## 4. Discussion

### 4.1. The Influence of Animal Models

The ideal animal model of lumbar disc degeneration would mimic the human degenerative process in terms of cellular, matrix, and biomechanical changes. Given the complex nature of disc degeneration, its multifactorial aetiologies and lengthy time-course, an animal model that exactly parallels the human condition is not feasible. Nonchondrodystrophoid animal species, in which there is persistence of notochordal cells, are less favourable as models due to a lower incidence of disc degeneration [67, 68]. Other important considerations are the quadruped posture of most animals and differences in disc shape and composition, which lead to biomechanical differences [69, 70]. Inherent difficulties in measuring pain and functional outcomes are important constraints. In addition to the abovementioned biological factors, there are economic and ethical constraints to consider when selecting an animal model [69, 70]

Methods of degeneration induction typically involve a disc injury such as chemical or mechanical nucleotomy or another disc lesion, which triggers a degenerative cascade within the insulted disc [25, 33, 37, 43, 49, 71, 72]. Although this may not be how degeneration typically starts in the human, it allows for the generation of a reproducible model bearing similarities to the human condition, which can then be used to assess disc repair [67, 68]. Conjecture regarding the ideal animal model should not be a hindrance to the progression of regenerative medicine [67, 68]. The perfect animal model of lumbar disc degeneration may not exist, and the authors cited in this review support the use of validated reproducible models, which can demonstrate the efficacy of cell based treatments. It is important, however, to interpret the results of such studies in the context of the animal model used.

By far the majority of studies identified in this review used smaller animal models, such as the rodent. There are several limitations to the use of such small animal models. The small disc dimensions in these animals are such that the very small cell volumes or scaffolds that are implanted are disproportionate to the volumes and construct specifications that are required in humans. The distance from the adjacent vertebral body or nutritional source is much closer than in humans. Moreover, these animals are nonchondrodystrophoid species, meaning that their notochordal cell population persists throughout life [73]. This differs from humans and chondrodystrophoid animals, such as the sheep, which are prone to disc degeneration following the disappearance of their notochordal cell population in early life [74]. Therefore, in studies performed in the smaller nonchondrodystrophoid animals, the validity of results must be questioned, as one cannot be sure whether the resident notochordal cells exert any regenerative effects, instead of, or in combination with, the transplanted cells. Treatments demonstrating efficacy in smaller animals should have safety and efficacy profiles demonstrated in larger chondrodystrophoid animal models with an aim for translation into human clinical trials, where appropriate [73].

### 4.2. Disc Chondrocyte Transplantation

The concept of transplanting healthy chondrocytes into a degenerate disc, in order to replace the depleted and senescent resident cell population, appears a sensible approach to disc repair. Intervertebral disc chondrocytes, such as NP cells, have been successfully isolated from intervertebral disc tissue, culture expanded, and used as a means to treat disc degeneration. 12 of 14 basic science studies and one clinical study, identified in this review, reported favourable outcomes following the transplantation of such cells into degenerate discs. Improved gross morphological and histological assessments, as well as increased proteoglycan content of the NP, are relatively consistent findings in the disc following chondrocyte transplantation. Moreover, preservation of disc height and increased MRI T2 signal point further to reconstitution of the disc extracellular matrix. Whether such preclinical outcomes correlate with clinically significant and durable improvements in humans has been only studied limitedly. The largest clinical trial to date, the EuroDISC study by Meisel et al. [75], demonstrated that clinical outcomes improved at 2 years.

Logically, it would appear that disc cells are ideal to use as cell based disc treatments; however, there are numerous limitations and impracticalities with the use of these cells clinically, the least of which not being that they must be harvested from disc tissue. Treatment is limited to patients requiring disc surgery; otherwise patients not undergoing disc surgery would require harvesting of cells from an adjacent disc, which would likely and inappropriately accelerate degeneration at that level [73]. In addition, the expense, expertise, and process of isolating, expanding, and storing disc cells under GMP (Good Manufacturing Practice) conditions will exclude such therapy from being available to most spinal surgery centres [76]. Moreover, disc cells, obtained from sequestered or prolapsed disc fragments, which are damaged discs whose cells may be effected by the degenerative process, may be inadequate for cartilage regeneration because of the heterogeneity of the tissue collected and low viability of the cells contained within these tissues [77].

The use of allogeneic chondrocytes, either obtained from surgical or from cadaveric donors, would potentially overcome the hurdles associated with the use of autologous disc cells. Although Nomura et al. [34] reported success with the use of allogeneic NP cells in the rabbit, with no evidence of immune or inflammatory response, the safety and efficacy of allogeneic transplantation of disc chondrocytes in the treatment of human disc degeneration has not been determined to date. Despite these limitations, the use of disc chondrocytes to treat disc degeneration is under investigation and is providing important insights into cell-based disc regeneration strategies as well as an understanding of disc pathology [75].

### 4.3. Transplantation of MSCs, MPCs, and Other Stem Cells

MSCs show exciting promise for disc repair and other tissue engineering strategies. MSCs can be isolated from numerous tissues including bone marrow, adipose tissue, and synovium [78–80], are reported to be nonimmunogenic [81, 82], and, unlike ESCs, lack the potential to undergo malignant transformation following transplantation [83]. MSCs possess the capacity for self-renewal, thus maintaining their undifferentiated phenotype in multiple subcultures, but when exposed to the appropriate stimuli they can undergo differentiation into cells of the mesenchymal lineage such as chondrocytes, osteocytes, tenocytes, and adipose cells. MSCs can be isolated from bone marrow aspirates by their ability to adhere to plastic culture plates, a technique that allows them to be separated from most of the other cellular components of the marrow that do not adhere. However, these cells consist of a heterogeneous population of cells including mixed MSC clones at various stages of differentiation together with contaminant mononuclear cells and fibroblasts. Nevertheless, it is evident from perspective of this review that MSCs isolated in such a fashion have the ability to repair damaged discs, at least partially.

The earliest uncommitted clonogenic populations of bone marrow stromal cells, designated mesenchymal progenitor cells (MPCs), can be distinguished by their expression of specific cell surface antigens including STRO-1, VCAM-1 (CD106), STRO-3 (tissue nonspecific alkaline phosphatase), STRO-4 (HSP-90b), and CD146 [84–86]. By using magnetic beads coupled to antibodies to these specific antigens it is possible to immunoselect particular clones from mixed cell populations. MPCs isolated in this manner, when expanded in culture, can generate cell banks of purified cells from a single donor, which retain extensive proliferative capacity and differentiation potential [85, 87, 88]. MPCs can be used in an allogeneic fashion [89, 90]. They have been produced so they can be used as “an off-the-shelf product” and are therefore well suited to the treatment of large numbers of patients, without the requirement for expensive GMP cell culturing capabilities at each and every treatment site. As allogeneic cells are taken from a young healthy donor, they are not subject to age related changes or other effects based on the protoplasm of the patient that can occur with the use of autologous cells [89].

Allogeneic MPCs have been demonstrated to reconstitute the disc extracellular matrix when injected into the degenerate ovine nucleus pulposus. Three months following the administration of the matrix degrading enzyme chondroitinase-ABC to ovine discs, animals injected with MPCs with a hyaluronic acid cell carrier demonstrated increased disc height, higher T2 MRI signal, and improved histological grading scores, with restoration of the extracellular matrix when compared with controls [40]. Our group is conducting further preclinical trials utilising such MPCs in ovine degeneration models, utilising a range of cell doses and cell carriers [89, 90]. Human allogeneic MPCs, isolated using the same methods, are currently under investigation in human clinical trials for the treatment of back pain due to degenerative disc disease [21].

MPCs or MSCs isolated from tissues are more accessible than intervertebral discs and appear to be the more practical alternative to disc cells for the treatment of disc disease. For these therapies to translate into clinical trials, it is important that they possess similar or greater efficacy than that of disc cell transplantation. Feng et al. [91] demonstrated that autologous MSCs and NPCs were comparable in terms of maintaining disc height and MRI T2 signal, production of proteoglycans (PGs), and expression of aggrecan and collagen II in a rabbit model. Allon et al. [63] similarly demonstrated comparable outcomes between allogeneic NP cells and MSCs, although MSCs had a lower survival time within the rat disc. This may have been due to less retention of the cells compared with bilaminar coculture pellets of MSCs and NPCs which were far more efficacious than either cell type when used alone. Acosta et al. [62] reported that allogeneic articular nondisc chondrocytes were far superior to MSCs in a porcine model; however, the poor viabilities and lack of regenerative potential demonstrated by their allogeneic bone marrow MSCs are not consistent with that of many of the other published MSC studies. These MSCs were derived from a different species; however the authors do not believe this was the cause of its poor efficacy. Furthermore, although these MSCs were shown to have chondrogenic differentiation potential, they were not characterized.

Once the safety and efficacy of intradiscal stem cell therapies are established, optimisation of the therapeutic intervention will need to be performed. Such optimisation will maximise the therapeutic and regenerative power that can be harnessed by these cells. What is the ideal cell dose to administer? What is the ideal cell carrier with which to administer the cells? Do repeated doses provide increased clinical benefit? Does the treatment provide benefit in the longer term? Answering these questions should be a focus of future research.

### 4.4. Cell Viability and the Maintenance of Cells within the Disc

Several studies reported very poor results with regard to the retainment of cells within the disc. Omlor et al. [49] reported only 9% of transplanted MSCs remaining in the disc 3 days after injection. Bertram et al. [25] found that 90% of cells leaked out when injected in aqueous form; however, this was reduced to 50% by coadministration with a fibrin glue. Vadalà et al. [57] found no evidence of GFP labelled MSCs in the disc at 9 weeks and concluded that the leakage of cells contributed to the development of peripheral osteophytes. Certainly these results require further studies to find methods that lead to greater retainment of cells within the discs space. To have some cell leakage may be unavoidable and perhaps highlights that only a portion of the transplanted cells need to engraft in the disc to impart benefit. Despite the above negative findings, the overwhelming majority of studies identified in this review that assessed survival of cells reported more favourable results. Transplanted NP cells were reported to be viable within the disc from 8 weeks to 8 months, whilst reported survival of MSCs ranged from 2 to 24 weeks following injection.

The issues of cell leakage and survival need to be considered when calculating the ideal cell dose to administer. Cell number considerations were not, however, discussed in the majority of studies. Serigano et al. [54] identified that the optimal dose of autologous MSCs in the canine was 1 × 10^6^ cells. From our group's own work, using allogeneic MPCs in an ovine model of disc degeneration, we found that an even lower dose of 0.1 × 10^6^ cells was the most efficacious [40]. A low dose of cells appears to be more beneficial due to the poor nutritional supply of the NP, which contributes to a ceiling effect, above which increased cell numbers cannot survive and may in fact be deleterious due to an accumulation of dead cells and waste products [40].

For transplanted cells to repair degenerate discs they must be retained within the disc long enough to exert an effect either by differentiation into chondrocytic cells which engraft within the disc or by the release of soluble factors that stimulate endogenous disc cells in a paracrine fashion. If cells leak out soon after implantation, their efficacy will be reduced or even abolished, leading to a negative response. Another important consideration after ensuring that cells remain within the disc is their survival in this hostile environment. The avascular, low glucose, low oxygen tension, low pH, and nutrient starved disc environment are some of the hostile factors transplanted cell must overcome in order for cells to survive following transplantation. For these reasons, numerous studies identified in this review specifically addressed this by assessing cell viability and survival within the disc but with conflicting results.

### 4.5. Mechanism of Action of Transplanted Cells

There are two predominant mechanisms by which transplanted cells are likely to impart their regenerative effects. Firstly, the cells can survive and proliferate in the target disc, acting as chondrocyte-like disc cells. These transplanted cells produce proteoglycan and collagen extracellular matrix components, thereby correcting deficiencies and restoring disc structure and function. Essentially these new healthy cells rescue the degenerate disc by replacing the depleted functional chondrocyte population. This is the likely mechanism of action of transplanted disc chondrocytes, which already have a chondrocytic phenotype. However, for undifferentiated stem cells such as MSCs or MPCs, the transplanted cells must first undergo chondrogenic differentiation within the disc if they are to assume a matrix producing phenotype. Henriksson et al. [42] demonstrated that human MSCs transplanted into the porcine disc differentiated into cells representing disc chondrocytes, expressing aggrecan, collagen II, and SOX-9. These transplanted cells produced disc matrix, and the level of matrix produced and cellular differentiation was increased with coadministration with a hydrogel carrier. Sakai et al. [52], Wei et al. [58], and Yang et al. [60] all reported site dependent differentiation of autologous MSCs toward NP phenotype, whilst Murrell et al. [48] demonstrated differentiation of olfactory neurosphere derived stem cells toward a NP phenotype. It is likely that factors released from resident disc cells, as well as contact with matrix components, stimulate the transplanted MSCs to undergo site-specific differentiation. Sobajima et al. [56] demonstrated synergism between NPCs and MSCs, with MSCs increasing PG production when cocultured with NPCs. Certainly there is evidence for chondrogenic differentiation following transplantation of MSCs; however, this is unlikely to be their sole mechanism of action.

The second potential mechanism of action is a paracrine effect, as transplanted cells can also act by releasing soluble trophic factors, which “kick start” or stimulate resident disc cells to produce disc matrix. MSCs and MPCs secrete anti-inflammatory cytokines and growth factors that are members of the Transforming Growth Factor (TGF) family [92]. These factors enhance NP cell synthesis of new matrix and suppress catabolic events triggered by mechanically mediated disc injury. Miyamoto et al. [92] demonstrated upregulation of Col2A1 (collagen type II) and downregulation of matrix degrading enzymes (TIMP-1, 2, MMP-2, 3, and 13) and inflammatory cytokines (TNF*α*, IL-3) in endogenous rabbit NP cells, following coculture with human MSCs. In addition, MSCs can stimulate the endogenous NP progenitors to proliferate or prevent them from undergoing apoptosis. Yang et al. [60] reported inhibition of disc cell apoptosis and disc matrix repair by transplanting autologous MSCs, adding further weight to the paracrine theory of mechanism of action.

Besides their regenerative properties, cellular therapies have another potential treatment pathway. The clinical benefits demonstrated in trials, such as reduction of pain, are unlikely solely attributable to the restoration of disc structure and mechanical function. Pain reduction is likely due to the potent immune-modulatory and anti-inflammatory properties that the transplanted cells possess, such as the regulation of Tumour Necrosis Factor *α* (TNF*α*) and other cytokines involved in the pain process.

### 4.6. Translation into the Clinic

The success of preclinical studies has led to several recent small clinical trials, publication of which also shows promising results. Yoshikawa et al. [23] first reported alleviation of both back and radicular symptoms following percutaneous administration of autologous MSCs in two elderly patients with degenerative disc disease [23]. Orozco et al. reported a study of 10 patients with chronic low back pain and degenerative disc disease treated with intradiscal administration of autologous MSCs [22]. 90% of participants reported clinical benefit, with significant decreases in pain and disability and improvement in quality of life. These studies were noncontrolled and nonrandomised, utilising a small number of heterogeneous patients, which detracts from the rigour of the successful results reported. An FDA of the USA approved phase-2 randomised controlled clinical trial, investigating the percutaneous, image-guided, intradiscal administration of allogeneic MPCs for the treatment of single level discogenic back pain in 100 patients, has now completed recruitment [93]. Results from this study are eagerly awaited as they will provide important data regarding the clinical benefit of stem cell therapies to treat disc disease.

## 5. Conclusion

Numerous studies have demonstrated success using cell-based therapies to treat disc disease, and these successes are in the early stages of translation into the clinic. Although not widely available, it is likely that stem cell therapies will become a treatment option for some patients with disc disease in the near future. Percutaneous stem cell mediated disc regeneration may bridge the gap between the two current alternatives for patients with low back pain, inadequate pain management at one end and invasive surgery at the other. Stem cell therapies to treat degenerative spinal conditions may not be the “miracle cure” that so many patients hope for. They are, however, likely to become part of the armamentarium that physicians can utilise to manage these patients.

The economics of cell-based therapies will need to be determined. Costs of current spinal treatments are enormous and increasing. For stem cell therapies to be utilised on a large scale, allogeneic administration of “off-the-shelf” stem cells, such as MPCs, is required. We consider the autologous route not viable for pragmatic and economic reasons. The costs of cellular therapies will need to be weighed up against cost savings in terms of increased work productivity and avoidance of more invasive and expensive procedures. Whether or not patients, health insurance, institutions, and public health providers will absorb the increased direct costs of cell-based therapies is yet to be established. Nonetheless, the promising results of studies so far should provide excitement to clinicians that manage patients burdened by this complex disease. This review provides a summation of the current landscape. These cell-based biological therapies are, for the first time, attempting to not only provide improvements in symptoms, but also attempt to restore biological structure and function of the disc.

## Figures and Tables

**Table 1:** Studies assessing the ability of disc derived and non-disc derived chondrocytes to regenerate lumbar intervertebral discs.

Author	Animal model	Degeneration model	Cells transplanted	Method of cell administration	Results/conclusions
Bertram et al. [25]	Rabbit	Nucleotomy and axial compression Amount of nucleus removed	Autologous NPCs	Percutaneous injection in fibrin gel	(i) 90% of cells leak out of disc space when injected in aqueous form (ii) Injection within fibrin-thrombin gel decreased leakage to 50% (iii) Intradiscal pressure limited cell survival

Ganey et al. [26]	Canine	Nucleotomy	Autologous disc chondrocytes	Intradiscal injection	(i) Transplanted cells remained viable and produced matrix similar in composition to native disc, including PGs (ii) Increased types II and I collagen (iii) Disc height maintained in cell treated group

Gorenšek et al. [27]	Rabbit	Nucleotomy	Autologous auricular cartilage derived chondrocytes	Intradiscal injection	(i) Production of hyaline cartilage in the NP (ii) Chondrocytes survived

Gruber et al. [28]	Rat	Partial annulotomy and nucleotomy	Autologous disc chondrocytes	Surgical implantation	(i) Transplanted cells remained viable producing matrix for up to 8 months (ii) Cells in AF had fibroblast appearance cells in NP had chondrocyte appearance

Huang et al. [29]	Rabbit	Nucleotomy	Allogeneic NPCs	NPC-seeded collagen*α*/ hyaluronan/ chondroitin-6-sulfate tri-copolymer constructs	NPC treated discs: (i) Increased MRI T2 signal (ii) Retard loss of disc height (iii) Produced cartilaginous matrix within NP (iv) Cells remained viable

Hohaus et al. [30]	Canine	Annular injury and partial nucleotomy	Autologous NPCs	Injection	NP cells: (i) Remain viable following implantation (ii) PG and ECM cartilage produced (iii) Types I and II collagen demonstrated (iv) Maintain disc height

Iwashina et al. [31]	Rabbit	Percutaneous aspiration of NP	Xenogeneic Human NPCs	Percutaneous injection	NPC treated discs: (i) Increased disc height (ii) Significantly less degeneration using morphological and histological analysis (iii) Increased proteoglycan synthesis (iv) Increased expression of aggrecan, versican, and collagen II

Luk et al. [32]	Rhesus monkey	Total discectomy	Allogeneic whole disc	Allogeneic whole disc surgical transplant	Fresh disc allografts: (i) Survive following transplantation (ii) Undergo severe degeneration after 24 months

Meisel et al. [15]	Canine	Discectomy	Autologous disc derived chondrocytes	Intradiscal injection	Transplanted cells: (i) Remain viable (ii) Produce matrix similar to normal disc (iii) Type I and II collagens demonstrated in regenerated intervertebral disc (iv) Maintained disc height

Nishimura and Mochida [33]	Rat	Percutaneous nucleotomy	Autologous NP tissue	Percutaneous injection	Implantation of NP tissue: (i) Delayed degenerative changes (ii) Preserved disc height

Nomura et al. [34]	Rabbit	Percutaneous aspiration of NP	Allogeneic NP cells and intact NP tissue	Percutaneous injection	(i) No immune or inflammatory response from allogeneic cell implantation (ii) Implantation of intact nucleus and NP cells reduced degeneration (iii) Increased type II collagen post implantation

Okuma et al. [35]	Rabbit	Percutaneous NP aspiration	Autologous NP cells	Percutaneous injection of NP cells cocultured with AF cells	Cell treated discs: (i) Delayed cell clustering (ii) Rate of degeneration slowed histologically (iii) Cells elaborated type II collagen

Ruan et al. [36]	Canine	Nucleotomy	Autologous NPCs	NP cells seeded onto L-lactic-co-glycolic acid (PLGA) scaffold	(i) Disc height, segmental stability, and MRI T2 signal preserved in NP treated discs (ii) PKH-26 labelled cells found in NP at 8 weeks

Sato et al. [37]	Rabbit	Vaporized using laser	Allogeneic annulus fibrosus cells	Annulus fibrosus cells cultured in atelocollagen honeycomb-shaped scaffold and labelled with PKH-26 fluorescent dye	Transplanted cells: (i) Prevented loss of disc height (ii) Remained viable at 12 weeks (iii) Produced hyaline cartilage

AF: annulus fibrosis, ECM: extracellular matrix, NP: nucleus pulposus, NPCs: nucleus pulposus cells, and PGs: proteoglycans.

**Table 2:** Studies assessing the ability of different types of stem cells or progenitor cells to regenerate lumbar intervertebral discs.

Author	Animal model	Degeneration model	Cells transplanted	Method of cell administration	Results/conclusions
Crevensten et al. [38]	Rat	Needle puncture	Allogeneic MSCs	Intradiscal injection of MSCs with 15% hyaluronan gel	MSCs: (i) Trend towards increased disc height (ii) Retained in disc, remain viable, and can proliferate for at least 28 days

Ganey et al. [39]	Canine	Partial nucleotomy	Non culture expanded autologous adipose derived stem cells	Intradiscal injection with HA	Transplantation of adipose MSCs improved: (i) MRI T2 signal at 12 months (ii) Disc histology assessment (iii) Increase collagen II expression

Ghosh et al. [40]	Sheep	Chondroitinase-ABC injection	Allogeneic Stro-3+ Mesenchymal Precursor Cells (MPCs)	Injection with hyaluronic acid (Euflexxa) carrier	MPCs + HA: (i) Restore disc height (ii) Improved MRI Pfirrmann scores (iii) Improved histological degeneration scores (iv) Restoration of extracellular matrix

Hee et al. [41]	Rabbit	Axial loading	Allogeneic bone marrow MSCs	Injection of MSCs combined with axial distraction	(i) MSCs increase disc height and improve histology scores (ii) MSCs Survive for 8 weeks

Henriksson et al. [42]	Porcine, minipig	Nucleotomy	Xenogeneic Human MSCs	Xenotransplantation of hMSCs with Puramatrix hydrogel carrier or F12 media suspension.	MSCs: (i) Survive in pig disc space for 6 months (ii) Differentiated into cells representing disc chondrocytes (iii) Improved MRI appearance in MSC/hydrogel treatment groups (iv) Combination of with Puramatrix hydrogel increased cell differentiation, matrix production and survival (v) At three and six months expressed SOX9, aggrecan, and collagen II

Hiyama et al. [43]	Canine	Nucleotomy	Autologous MSCs	Percutaneous injection of MSCs infected with AcGFP1 retrovirus vector.	MSCs: (i) Increased disc height and MRI T2 signal (ii) Increased production of proteoglycans (iii) Improved histological structure including AF (iv) Proportion of FasL-positive cells increased following MSC injection

Ho et al. [44]	Rabbit	Percutaneous needle puncture	Autologous MSCs	Intradiscal injection of BrdU-labelled MSCs	MSCs: (i) Found in disc at 16 weeks post injection (ii) Discs injected at 6 months post nucleotomy less degenerate than controls but not returned to baseline (iii) Increased PG in posterior inner annulus (iv) Did not restore disc height (v) Only partial arrest possible following administration and more effective at later point of degeneration

Hohaus et al. [30]	Canine	Annular injury and partial nucleotomy	Autologous adipose derived MSCs	Intradiscal injection	Adipose MSCs (i) Remain viable in disc (ii) Maintain disc morphology, disc height, and MRI T2 signal (iii) HA alone insufficient to prevent degeneration

Jeong et al. [45]	Rat	Annular injury	Xenogeneic human MSC	Intradiscal injection	MSCs: (i) Maintain disc height and T2 signal (ii) Restore AF structure (iii) Survive for 2 weeks after injection but not 4 weeks

Jeong et al. [46]	Rat	Needle injection	Xenogeneic adipose derived human MSCs	Intradiscal injection	MSCs: (i) Less loss of disc height following injection (ii) Restore T2 MRI signal (iii) Restore AF structure (iv) Upregulate collagen 2 and aggrecan

Miyamoto et al. [47]	Rabbit	NP aspiration	Autologous synovial MSCs	Intradiscal injection	MSCs: (i) Identified in NP at 24 weeks (ii) Preserve disc height (iii) Preserve MRI T2 signal for 6 weeks (iv) Preserve NP histological structure (v) Increase expression of collagen-II

Murrell et al. [48]	Rat	NP aspiration	Xenogeneic human olfactory neurosphere-derived stem cells	Intradiscal injection	(i) 70% cells identified in discs (ii) Cells assumed NP cell like phenotype

Omlor et al. [49]	Porcine	Partial nucleotomy	Autologous Bone marrow MSCs	Injection of MSCs transfected with Rv-eGFP within fibrin glue	(i) After 3 days only 9% of injected cells remained in disc

Prologo et al. [50]	Porcine	Needle biopsy of disc	Xenogeneic human MSCs	Xenogenic percutaneous administration of iodine-124 2′-flouro-2′ –deoxy-1B-D-arabinofuranosyl-5-iodouracil –labeled hMSCs	(i) PET-CT confirmed cells in NP on day 0 and day 3 following injection. (ii) Immunohistological staining at 15 days confirmed presence of cells in treated discs

Sakai et al. [51]	Rabbit	Nucleotomy – NP aspiration.	Autologous MSCs	MSCs embedded in atelocollagen hydrogel	MSCs: (i) Preserved histological structure (ii) Retained and proliferated in disc (iii) Increased PGs on histological staining

Sakai et al. [52]	Rabbit	Nucleotomy – NP aspiration.	Autologous bone marrow MSCs	GFP labelled MSC injection	(i) MSCs present in NP at up to 48 weeks (ii) GFP positive cells expressed collagen II, aggrecan, suggesting site dependent differentiation (iii) MSCs increased PG content of NP to baseline (iv) Increased collagen II and aggrecan mRNA, decreased collagen I following MSC injection

Sakai et al. [53]	Rabbit	Nucleotomy – NP aspiration.	Autologous bone marrow MSCs	MSCs embedded in atelocollagen hydrogel	(i) MSCs increased disc height and MRI T2 signal (ii) MSCs preserve histological structure, including AF (iii) Restoration of PGs suggested from immunohistochemistry and gene expression

Serigano et al. [54]	Canine	NP Aspiration	Autologous bone marrow MSCs	Intradiscal injection	(i) MSCs significantly increase DHI and MRI T2 signal (ii) 10^6^ and 10^7^ cell doses showed improved NP and inner annular histological structure (iii) 10^5^dose group had more degenerative changes (iv) 10^6^ dose group had less apoptosis than 10^5^ or 10^7^ groups (v) 10^6^ dose group had more live cells at 16 weeks compared to other groups

Sheikh et al. [55]	Rabbit	Needle puncture	Xenogeneic murine ESCs were cultured with cis-retinoic acid, transforming growth factor beta, ascorbic acid, and insulin-like growth factor	Intradiscal injection	(i) Discs treated with ESCs demonstrated increased population of new notochordal cells

Sobajima et al. [56]	Rabbit	Normal discs	Allogeneic MSCs	Injection with MSCs retrovirally transfected with lacZ marker gene.	(i) MSCs detected up to 24 weeks following transplantation. (ii) No inflammatory response observed in discs following MSC injection (iii) At 24 weeks more cells located in transition zone and inner AF, taking on more spindle shaped appearance (iv) Synergism with NPCs and MSCs to increase GAG production, most at 75 : 35 MPC/MSC ratio

Vadalà et al. [57]	Rabbit	Needle Stab	Allogeneic bone marrow MSCs	Intradiscal injection	(i) No evidence of regeneration at 9 weeks on MRI (ii) X-ray demonstrated osteophyte formation in treated discs (iii) No cells found in NP using GFP label

Wei et al. [58]	Rat	Nil	Xenogeneic human bone marrow MSCs – CD34− (MSCs) and CD34+ (Haemopoeitic cells) bone marrow cells	Intradiscal injection	(i) CD34− cells (MSCs) remain in NP for 42 days (ii) CD34+ cells not visible after day 10 (iii) CD34− cells expressed CTO/collagen II or CTO/Sox-9 indicating chondrocytic phenotype differentiation (iv) No inflammatory cells visible

Yang et al. [59]	Mouse	Annular puncture	Allogeneic bone marrow MSCs	Intradiscal injection	MSCs: (i) Preserve NP and AF structure up to 24 weeks (ii) Preserve disc height (iii) PGs upregulated (iv) Decrease in Col2a1, aggrecan and Sox9 arrested (v) GAG/DNA increased (vi) Underwent chondrocytic differentiation (vii) Increased notochordal cells suggesting MSCs promote NCC survival and proliferation (viii) Cells survive 24 weeks using GFP labelling

Yang et al. [60]	Rabbit	Needle puncture and nucleotomy	Autologous MSCs	Injection of MSCs with pure fibrinous gelatin-transforming growth factor-beta1 (PFG-TGF-beta1)	(i) MSCs inhibited apoptosis (ii) MSCs slowed the rate of loss of DHI and increased T2 signal at 12 weeks (iii) Increased type II collagen in MSC treated group

Zhang et al. [61]	Rabbit	Normal discs	Allogeneic bone marrow MSCs	Injection of LacZ labelled MSCs	(i) MSCs survive in disc (ii) MSCs increase expression of Type II collagen and PGs

AF: annulus fibrosis, DHI: disc height index, ESCs: embryonic stem cells, GAG: glycosaminoglycan content, GFP: green fluorescent protein, HA: hyaluronic acid, MSCs: mesenchymal stem cells, MPCs: mesenchymal precursor cells, NP: nucleus pulposus, NCC: notochordal cell, NPCs: nucleus pulposus cells, and PG: proteoglycans.

**Table 3:** Studies comparing the efficacy of MSCs and chondrocytes to regenerate lumbar intervertebral discs.

Author	Animal model	Degeneration model	Cells transplanted	Method of cell administration	Results
Acosta Jr et al. [62]	Mini pig	Nucleotomy	Allogeneic juvenile articular nondisc chondrocytes (JCs) and allogeneic bone marrow MSCs	Injection of MSCs or chondrocytes in fibrin carrier	(i) Higher GAG and DNA content in JC group (ii) JC group higher cartilage/collagen II production (iii) JC cells viable at 12 months (iv) MSCs not viable at 3 months and no evidence of PG production

Allon et al. [63]	Rat	Nucleotomy	Allogeneic bone marrow MSCs and allogeneic NPCs	Bilaminar coculture pellets (BCPs) of MSCs and NPCs in a fibrin sealant	(i) Increased disc height in BCP group – combined MSC + NPC (ii) PG produced by BCP (iii) Less viability of MSCs in disc compared to NPCs, otherwise no differences between MSCs and NPCs

Feng et al. [64]	Rabbit	Nucleotomy	Autologous bone marrow MSCs and autologous NPCs	Intradiscal injection	MSCs and NPCs comparable in (i) Maintaining disc height and T2 signal (ii) Maintaining gene expression of aggrecan and collagen II (iii) Producing PGs

BCP: bilaminar coculture pellets, GAG: glycosaminoglycan content, JC: juvenile chondrocytes, MSCs: mesenchymal stem cells, NPCs: nucleus pulposus cells, and PG: proteoglycans.

**Table 4:** Clinical studies utilising cell-based therapies to treat human lumbar disc degeneration.

Author	Clinical details	Cells transplanted	Method of cell administration	Results	Level of evidence
Haufe et al. [65]	10 patients with low back pain due to degenerative disc disease	Autologous bone marrow haematopoietic precursor stem cells (HSCs).	Percutaneous injection with concurrent hyperbaric oxygen therapy	(i) No improvement in back pain in any patient (ii) 80% of patients underwent surgical intervention within 1 year	(i) 3

Meisel et al. [15]	28 patients undergoing microdiscectomy with back pain (*EuroDISC* study)	Autologous culture expanded disc derived chondrocytes	Percutaneous injection 12 weeks following microdiscectomy	(i) Patients receiving cell transplantation had reduced back pain at 2 years (ii) Increased MRI T2 signal of treated and adjacent discs	(i) 1

Orozco et al. [22]	10 patients with low back pain and radiological evidence of degenerative disc disease	Autologous MSCs	Percutaneous injection	(i) Clinical improvement in back pain, leg pain and disability (ii) Increased MRI T2 signal (iii) Disc height not recovered	(i) 3

Yoshikawa et al. [23]	2 patients with back pain and sciatica, with radiological evidence of lumbar canal stenosis and disc disease	Autologous bone marrow MSCs	Percutaneous injection within collagen sponge	(i) Increased MRI T2 signal (ii) Less instability (iii) Clinical improvement in both patients	(i) 3

HSCs: haematopoietic precursor stem cells, MSCs: mesenchymal stem cells.
